# All in the Family? Age, Period, and Cohort Differences in Kinship Ties Among Older U.S. Adults

**DOI:** 10.1093/geroni/igab046.1523

**Published:** 2021-12-17

**Authors:** Ellen Compernolle, Khoa Phan Howard, Eric Hedberg

**Affiliations:** 1 NORC at the University of Chicago, Chicago, Illinois, United States; 2 Northwestern University, Evanston, Illinois, United States; 3 Abt Associates, Chicago, Illinois, United States

## Abstract

In general, older adults’ social networks—characteristics of which (e.g., size, type, frequency) have been linked to important health and well-being outcomes--tend to be kin-centered, although this has changed over time. Disentangling these changes, however, is difficult given typical mobility decline and shrinking networks in old age (age), the rapid social and demographic changes that occurred during the 20th century (cohort), and, in recent decades, the 2008 Recession and technological advances (period). This study uses data from the National Social Life, Health, and Aging Project (NSHAP), a nationally representative sample of older adults (ages 57-85; 2005-2016), to examine patterns in older adults’ social networks, with particular emphasis on the role that family plays. Specifically, we ask: 1) Have older adults’ social networks become less kin-centered over the past decade (2005-2016)? 2) Are they less kin-centered among younger cohorts? And 3) Does the recession explain part of these period effects? We find that, between 2005 and 2016, family still comprises the majority of older adults’ social networks, although their network size and range have grown larger and become less family-centric. They also report fewer close family members and friends, living with fewer family members, and less frequent interaction with network ties. Results from multi-level regression models suggest that age, and to a much lesser extent, cohort, plays a key role in many of these changes, although this varies between the first and second 5-year intervals of data collection, underscoring older adults’ adaptivity to current social and economic circumstances.

